# Active Surveillance for Renal Angiomyolipoma Less Than 4 Centimeters: A Systematic Review of Cohort Studies

**DOI:** 10.7759/cureus.22678

**Published:** 2022-02-28

**Authors:** Mohamed Zeid, Hani Sayedin, Nauman Nabi, Mamoun Abdelrahman, Prem Thomas Jacob, Bassem Alhadi, Subhasis Giri

**Affiliations:** 1 Urology, University Hospital Limerick, Limerick, IRL; 2 Urology, Warrington and Halton Teaching Hospitals National Health Service (NHS) Foundation Trust, Warrington, GBR; 3 Emergency Department, Mater Misericordiae University Hospital, Dublin, IRL

**Keywords:** kidney disease, conservative approach, benign renal mass, aml treatment, renal angiomyolipoma

## Abstract

The aim of this review is to evaluate the current evidence regarding the best management in terms of active surveillance of angiomyolipoma (AML) cases less than 4 cm, particularly the optimal timing of active surveillance. In addition, we aimed to describe their initial size, clinical presentation, and growth rates. The present systematic review included prospective and retrospective studies that evaluated and followed up patients with AML through active surveillance. Studies were retrieved through an online bibliographic search of the Medline database via PubMed, SCOPUS, Web of Science, and Cochrane Library from their inception to January 2022. Seven studies were included in the present systematic review. Concerning the active surveillance protocol, only four studies describe the frequency of active surveillance and the utilized imaging modality. Some studies followed up lesions by ultrasound annually for two to five years, while other studies followed-up patients twice for the first year, then annually for a median follow-up period of 49 (9-89) months. The used modalities were ultrasound, CT, and magnetic resonance imaging (MRI). Notably, the incidence of spontaneous bleeding was consistent across the included studies (ranging from 2.3 - 3.1%), except for one study which showed an incidence rate of 15.3%. In terms of the need for active treatment, the rate of active treatment was slightly higher in some studies than the others. However, this variation could not be considered clinically relevant to favor one surveillance strategy over the other. We concluded that active surveillance is the first line of management in all small asymptomatic ALMs. ALMs less than 2 cm do not require active surveillance. The current published literature suggested that active surveillance for two years may provide the same benefits as a five-year surveillance strategy, with fewer radiation hazards and less socioeconomic burden.

## Introduction and background

One of the most prevalent solid benign renal masses is angiomyolipoma (AML). The majority of AMLs are sporadic, with varying amounts of blood vessels, smooth muscle, and fat, while 10% of AMLs are hereditary and linked to tuberous sclerosis [1]. In patients with tuberous sclerosis, AMLs have a tendency for faster growth rate, higher rates of spontaneous bleeding, more aggressive behavior, and a higher incidence of intervention [2]. The prevalence of sporadic AML is believed to be 0.44% in the general population, with the majority of cases being discovered incidentally in asymptomatic patients [3, 4]. Incidental renal tumors are being found as a result of the increased use of body imaging by computed tomography (CT), ultrasound, and magnetic resonance imaging (MRI) [5].

The majority of renal AMLs are asymptomatic; however, abdominal and flank pain and haematuria are typical clinical symptoms [6]. Spontaneous growth and bleeding can occur despite the benign nature of these tumors, especially in tumors larger than 4 cm [7-9]. Active surveillance for small AML is recommended by the European Association of Urology [10]. Yet, there is no agreement on the best scanning modality or interval between scans [11]. AML larger than 4 cm are more likely to require surgical intervention; however, this size cut-off point has lately been questioned [12-15].

Selective arterial embolization, mechanistic targeting of rapamycin (mTOR) inhibitors, and partial nephrectomy or nephrectomy are all options for treating AML [2, 16]. Treatment is recommended in well-selected cases, such as big tumors, those in women of child-bearing age, and if follow-up or access to emergency care is insufficient [10]. Many studies proposed that regardless of initial size, the majority of incidentally found AMLs develop slowly, stay asymptomatic, and hence do not require intense follow-up or early intervention.

This systematic review aimed to evaluate the current evidence regarding the best management in terms of active surveillance of AML cases less than 4 cm, particularly the optimal timing of active surveillance. In addition, we aimed to describe their initial size, clinical presentation, and growth rates.

## Review

Materials and methods

We followed the standards recommended by the Cochrane Collaborative Group [17] and PRISMA checklist [18] to prepare the present systematic review and meta-analysis.

Literature search and eligibility criteria

Studies were retrieved through an online bibliographic search of Medline database via PubMed, SCOPUS, Web of Science, and Cochrane Library from their inception in January 2022. A combination of the following keywords were used for the bibliographic search: "renal angiomyolipoma", "AML", "surveillance", "active surveillance", "follow-up", "management", and "management protocol". We included prospective and retrospective studies that were published in the English language and followed up patients with AML through active surveillance. There were no restrictions regarding the country or the year of the study. Only studies with reliable data regarding the outcomes of AML at the end of active surveillance. We excluded duplicate datasets, review articles and other forms of non-original publications, and thesis. The online search was complemented by manual searching of the references of eligible studies.

Screening and data extraction

All retrieved records were imported into Endnote X8 program (Thompson Reuter, USA) for duplicates removal. The screening of the unique records passed through two stages: titles and abstracts screening, and full-text screening of the abstracts deemed eligible for the present review. Each step was done by two reviewers independently according to the predetermined criteria. Disagreements at any stage were resolved by consensus. Two reviewers independently extracted the following data from eligible studies data collection form: study design, main findings, the sample size, characteristics of the patients, follow-up duration, active surveillance protocol, growth rate, need for active intervention, and incidence of hemorrhage and rupture.

Results

**Figure 1:**
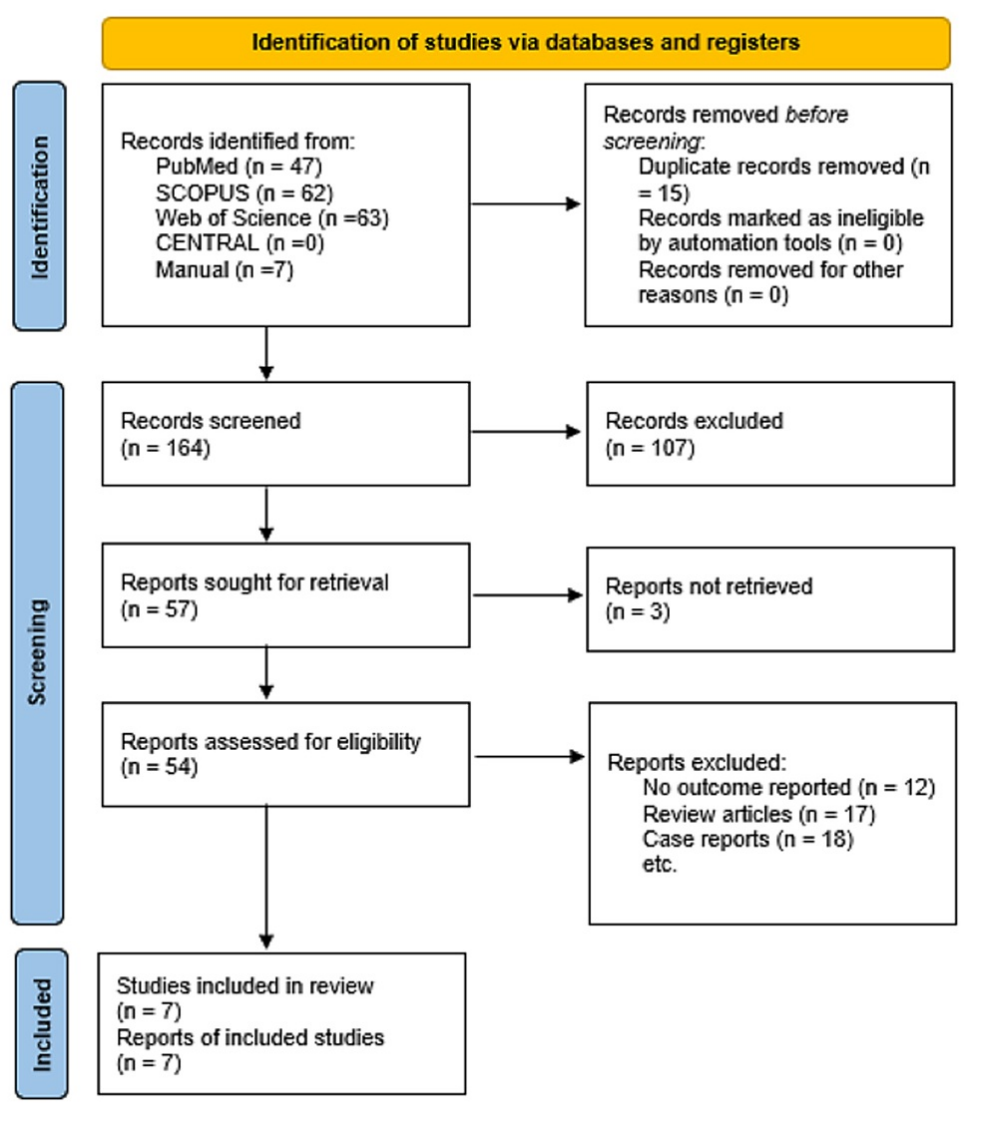
Indications of studies via databases and registers

One-hundred and sixty unique records were initially screened. Of them, 57 full texts were downloaded and screened for potential inclusion in the present review. Overall, seven studies were included in the present systematic review. The PRISMA flowchart is shown in Figure 1.

Table 1 shows the summary characteristics of the seven included studies. Four studies were retrospective chart reviews and all studies included patients with a diagnosis of AML, either incidentally or upon clinical presentation. The sample size of the included studies ranged from 45 to 587 AML patients. The characteristics of the included patients are presented in Table 2.

**Table 1:** The characteristics of the included patients

Authors (Year)	Country	Study Design	Population	Number of patients	Main Findings
Bhatt et al. (2016) [15].	Canada	Retrospective study	Patients who underwent CT or ultrasound	447	Lesions >4 cm do not require early intervention based on size alone. The majority of untreated AMLs (>92%) had not grown at a median follow- up of 43 months.
Chan et al. (2018) [11].	UK	Prospective study	Patients with sporadic angiomyolipoma	187	The majority of sporadic angiomyolipomas are small and do not grow. mean growth rate of 0.13±0.88 mm/year. Surveillance should be performed for those greater than 20 mm, with five-yearly ultrasound scans for 21–29 mm, and two-yearly surveillance for 30–39 mm tumours.
MacLean et al. (2014) [19].		Prospective study	Patients with renal angiomyolipomas	135	Small, solitary AMLs ( 20 mm) do not require follow-up due to their low probability of growth. Patients with multiple AMLs and younger patients require closer monitoring due to their comparatively greater AML growth rate. Ultrasound-detected AMLs with an extra-renal component may require computed tomography (CT) to confirm their size
Ouzaid et al. (2014) [20].	USA	Retrospective study	Patients diagnosed with AMLs on computed tomography (CT) who were managed with AS	130	Patients who underwent delayed intervention were more likely to present with a higher body mass index, larger tumours and symptomatic disease. Tumour size and symptoms were independently associated with discontinuation of AS. Selective angioembolization was the first-line option used for AT after AS was discontinued.
Mues et al. (2010) [14].	USA	Prospective study	Patients with sporadic AMLs	45	After a median follow-up of 54.8 months, there was a mean growth rate of 0.088 cm/year. AS for AMLs is associated with a slow and consistent growth rate (0.088 cm=year), typically has minimal morbidity, and is a reasonable option in selected patients. Symptomatic presentation and size (>3 cm) are not predictive for necessitating an invasive procedure.
Lee et al. (2019) [21].	Taiwan	Retrospective study	Patients who were diagnosed with renal angiomyolipoma by ultrasonography, CT, or MRI.	587	The optimal cut-off point on the ROC curve for predicting SAML tumor hemorrhage was 7.35 cm. A larger tumor size, younger patient’s age and higher BMI value correlated with a higher risk of tumor hemorrhage. For tumor sizes less than 7.35 cm, active surveillance or TAE for hemorrhage prevention were recommended. Surgical management should be considered for patients with tumors larger than 7.35 cm, symptomatic and progressive AML, or suspicious EAML.
Dorin et al. (2014) [22].	USA	Retrospective study	Patients with renal masses who were diagnosed either incidentally or upon clinical presentation using US, CT, or MRI.	114	Renal masses under active surveillance grew slowly, and had a low incidence of requiring surgical intervention and progression. Mean maximal tumor diameter growth rate for all renal masses was 0.72±3.2. Solid enhancing masses grew slowly, and were more likely to trigger intervention. Active surveillance should be considered for selected patients with small renal masses

**Table 2:** Summary characteristics of the included studies * Not reported

Authors (Year)	Age –average	Male %	% Sporadic AML	Size average (cm)	Size range	Size other reports	Presentation		
							Asymptomatic	Symptomatic	Incidental presentation (%)
Bhatt et al. (2016) [15].	58.1 (18.5–90.3)	19.90%	NR	89.5% ≤ 4 cm, 10.5% > 4 cm	NR	NR*	0%	9.20%	90.8
Chan et al. (2018) [11].	61 (20–89)	19.80%	100%	0.9 cm	0.3–8.6 cm	NR	NR	NR	NR
MacLean et al. (2014) [19].	≤20mm: 50 years;	≤20mm: 17.9%;	NR	NR	NR	NR	NR	NR	NR
	>20 to <40mm: 45 years;	>20 to <40mm: 20%;							
	≥40mm: 49 years	≥40mm: 25%							
Ouzaid et al. (2014) [20].	53.3 (16.5)	77.70%	100%	70.8% < 4 cm, 29.2% ≥ 4 cm		NR	0%	21.50%	78.5
Mues et al. (2010) [14].	59 (40–75)	23%	100%	1.7 cm	0.3–8.0 cm	NR	NR	15.38%	83.9
Lee et al. (2019) [21].	51(0.4 - 100)	22.70%	87.40%	5.8	4.7(0.3-32.4)	TSCAML =2.7(0.3-8.4) with average of 5.9 cm	50.50%	NR	NR
						EAML:10.5(1.6-21) with average of 9.5 cm			
Dorin et al. (2014) [22].	69.1 (20.7-89.7)	47.36%	NR	NR	NR	NR	NR	NR	NR

The average age of the included patients ranged from 45 to 69.1 years old, with notable female predominance. Sporadic AML accounted for 87.4 to 100% of the lesions in the included studies. The average size of all lesions ranged from 0.3 to 32.4 cm; however, more than two-thirds of the lesions within the included studies were less than 4 cm. Across the included studies, 78.5 - 90% of the cases were discovered incidentally.

Concerning the active surveillance protocol, only four studies describe the frequency of active surveillance and the utilized imaging modality. In Chan et al., lesions with size <2 cm were not followed up, lesions with size 2-3 cm were followed by ultrasound annually for five years, and lesions with size 3-4 cm were followed by ultrasound annually for two years. While MacLean et al., followed patients for 12 months using ultrasound and computed tomography (CT). In Ouzaid et al., patients were followed up by CT semi-annually for the first year, then annually for a median follow-up period of 49 (9-89) months. Finally, Dorin et al. stated that the frequency of active surveillance ranged from once per two to once per 17 months; the used modalities were ultrasound, CT, and MRI, (Table 3).

**Table 3:** Summary of active surveillance protocol

Authors (Year)	Follow up Available	Frequency	Modality	Duration, months (range)
Bhatt et al. (2016) [15].	Yes	Not reported	Not reported	43 (14–144)
Chan et al. (2018) [11].	Yes	<2 cm: no FU	US	30 (8.66–51.34)
		2–3 cm: 5 yr		
		3–4 cm: 2 yr		
		>4 cm: consider surgery		
MacLean et al. (2014) [19].	Yes	12 mo	US and CT	21.8 (6–85.3)
Ouzaid et al. (2014) [20].	Yes	1st follow up/ 6 mo	CT	49 (9–89)
		2nd follow up / 6 mo		
		Then yearly		
Mues et al. (2010) [14].	Yes	NR	Radiological	54.8 (0.2–211.7 ),
Lee et al. (2019) [21].	Yes	NR	NR	NR
Dorin et al. (2014) [22].	Yes	6 (2-17)	CT, MRI and US imaging	50.4 ± 30.6

Notably, the incidence of spontaneous bleeding was consistent across the included studies (ranging from 2.3 - 3.1%), except for Lee et al., which showed an incidence rate of 15.3%. In terms of the need for active treatment, the rate of active treatment was slightly higher in Bhatt et al., (5.6%) and Ouzaid et al., (13.1%), than Chan et al. (2.8%), MacLean et al. (2.2%), and Mues et al. (13.1%). However, this variation could not be considered as clinically relevant to favor one surveillance strategy over the other (Table 4).

**Table 4:** Outcomes of the included patients

Author (Year)	Growth Rate (cm/year)	Spontaneous bleeding (%)	Active treatment (%)	Indication analysis for active treatment and size cut-off	Recurrence rate
Bhatt et al. (2016) [15].	0.02	2.7	5.6	NR	NR
Chan et al. (2018) [11].	0.1	0	2.8	NR	NR
MacLean et al. (2014) [19].	0.015	2.2	2.2	NR	NR
Ouzaid et al. (2014) [20].	NR	3.1	13.1	NR	NR
Mues et al. (2010) [14].	0.08	2.3	6.7	NR	NR
Lee et al. (2019) [21].	NR	15.30	56.10	7.35 cm	NR
Dorin et al. (2014) [22].	0.75±4.4	NR	NR	NR	NR

Discussion

The extensive use of abdominal imaging has increased by the discovery of sporadic renal AMLs, which account for 80% of all cases [23, 24]. Despite their benign nature, they can develop to large sizes and can occasionally induce spontaneous bleeding [25, 26]. Because the natural history of sporadic AMLs is unknown, current recommendations encourage surveillance of smaller tumors but do not guide the appropriate timing or mode of follow-up [21]. This systematic review summarizes the current evidence regarding the size, growth rate, clinical characteristics, and surveillance protocols of ALMs.

Regarding the size of ALMs, the majority of the included studies reported a small size of ALM (<4 cm) in most patients. Bhatt et al. reported that about 90% of the patients had ALMs less than 4 cm [15], while Ouzaid et al., found that the percentage was 70.8% [20]. Similarly, Chan et al and Mues et al. reported a small size of ALMs in their patients 0.9 cm and 1.5 cm, respectively [11, 14]. On the other hand, Lee et al. mentioned that the average size of ALMs was 5.8 cm. They also mentioned that the average size of tuberous sclerosis complex associated ALM (TSCAML) and epithelioid AML (EAML) was 5.9 cm and 9.5 cm, respectively [21]. In the study of MacLean et al., the majority of patients (74.8%) had ALMs less than 2 cm, 16.3% had ALMs between 2-4 cm, and 8.9% had ALMs larger than 4 cm [19]. These findings indicate that more sensitive imaging technology is detecting a higher number of small AMLs. This emphasizes the significance of adopting an evidence-based methodology for monitoring these more prevalent lesions.

In terms of the growth rate, the highest growth rate was reported by Dorin et al. (0.75±4.4 cm/year) [22]; however, many other studies reported slower growth rate as in Chan et al. (0.1 cm/year) [11], MacLean et al. (0.015 cm/year) [19], Bhatt et al. (0.02 cm/year) [15], and Mues et al. (0.08 cm/year) [14]. Dorin et al. suggested that the growth rate depends mainly on the type of lesion. For example, the most aggressive type from their experience was the epithelioid variant of AMLs, followed by solid masses, which showed an intermediate growth rate, with a high tendency to progress to intervention during active surveillance. On the other hand, they observed that complex cystic lesions showed essentially zero growth over the time course of the study period [22]. Chan et al. found that there was no significant association between the initial size of ALMs and growth rate [11], which was also reported by Bhatt et al., who reported comparable findings when compared lesions ≤4 cm and >4 cm in terms of growth rate (p=0.86) [15]. On the other hand, MacLean et al. found that larger initial tumor size was associated with a higher growth rate (p <0.005). Small AMLs appear to be less likely to grow compared to large ones. As a result, most small AMLs are unlikely to become large enough to cause symptoms or consequences [19]. Therefore, with the exception of young patients or those with hereditary subtypes, who are more prone to display growth, the follow-up of small AMLs can be regarded unnecessary.

The majority of renal AMLs are asymptomatic; however, abdominal and flank pain and haematuria are typical clinical symptoms [27]. The most serious complication of ALMs is the spontaneous bleeding resulting from lesion rupture due to its increasing size [28-30]. It might induce hemodynamic instability and shock in some instances [31]. As a result, predicting the likelihood of AML spontaneous bleeding is a critical clinical concern. Nevertheless, the rate of spontaneous bleeding among the included studies was low, ranging from 0% to 3.1% [11, 14, 15, 19-20,22]. Only Lee et al. [21], reported a high rate of spontaneous bleeding (15.3%). Therefore, they further studied the difference between patients with positive bleeding and those without. Their findings demonstrated that patients with spontaneous bleeding were associated with younger age (p=0.004), higher BMI (p=0.046), larger tumour size (p<0.001), lower hemoglobin (p<0.001), and higher creatinine (p=0.048). In addition, the rate of spontaneous bleeding was higher in females compare to males (68.3% vs. 31.7%). Likewise, the mean tumor sizes of hemorrhaging and non-hemorrhaging AML rates in Prando's research were 11.4 cm and 5.0 cm, respectively, implying that the larger the tumor, the greater the chance of bleeding [32]. Obesity has been associated with a higher risk of renal tumor, especially in women [33, 34]. A high BMI is a strong independent risk factor for renal cancer in both men and women [35, 36]. However, in ALMs the role of obesity is yet unknown. Regarding the association between tumor size and risk of bleeding, Lee et al. [21], performed a ROC analysis to identify the optimal cut-off point, which was 7.35 cm, meaning that patients with ALMs were larger than 7.35 cm were at higher risk of developing spontaneous bleeding. Hence, they recommended performing active surveillance for stable patients with ALMs less than 7.35 cm and application of intervention in case of ALM larger than 7.35 cm. Major bleeding was common in sporadic AMLs with a diameter of >6 cm, according to a study by Kuusk T et al. They indicated that AMLs with a diameter of less than 6 cm should be treated conservatively [37].

The intervention rate varied among the included studies, ranging from 2.2% to 56.1% [11, 14, 15, 19-22]. In the study of Bhatt et al. [15], interventions were carried out in 5.6%. Patients who required intervention were associated with younger age (p=0.002), presence of tuberous sclerosis complex (p<0.0001), symptomatic presentation (p<0.0001), larger size >4 cm (p<0.0001), and higher growth rate >0.25 cm/year (p=0.03). In the study of Lee et al. [21], the rate of intervention was 56.1%, which is very high compared with other studies; however, it could be explained by the relatively large initial size of ALMs of their included patients (6 cm), compared with others. In the study of Mues et al. [14], the rate of intervention was 6.7%. They demonstrated that the active surveillance experienced three failures, needing intervention. Two patients needed selective embolization due to retroperitoneal bleeding. For a centrally placed tumor, one patient had a considerable growth rate (0.7cm/year) and required radical nephrectomy. About 13% of the patients in the study of Ouzaid et al. [20], discontinued active surveillance and underwent active treatment. Multivariate analysis showed that tumor size and clinical symptoms were the significant independent predictors of active surveillance failure and the necessity of active treatment. They also concluded that selective angioembolization was the first-line option used for active intervention T after active surveillance, followed by partial nephrectomy.

Regarding the best strategy for surveillance, there is no consensus among the included studies. Chen et al. [11], recommended that all lesions larger than 2 cm should undergo active surveillance, lesions between 2-3 cm should be evaluated every five years, and lesions larger than 3 cm should be evaluated every two years. In the scheduled visits, if the size of the lesion decreased or did not change, they discharge the patients, but if it increased, they continued the surveillance. For Droin et al. [22], the average follow-up period was 4.2±2.6 years. MacLean et al. [19], followed their patients every 12 months for 21.8 (6-85.3) months. Despite the variation in the scheduled visits and follow-ups, the majority of these studies reported comparable findings, demonstrating that increasing the period of surveillance from every five years to every two years is not necessary and does not add that much to the patient management plan, but, on the other hand, it increases the risk of radiation exposure and the increases the burden on healthcare systems due to the high cost of these diagnostic modalities [38, 39].

We acknowledge that our study has some limitations including the small sample number of included studies and the retrospective nature of some of them. In addition, the moderate risk of bias in the included studies may hinder the quality of generated evidence. Further, we could not perform a meta-analysis due to the lack of relevant data.

## Conclusions

AML have variable sizes and presentations; however, asymptomatic small sizes AML are more prevalent than large symptomatic. Active surveillance is the first line of management in all small asymptomatic AML. AML less than 2 cm might not require active surveillance. Young patients with high BMI and large size AML should be evaluated for the risk of rupture and spontaneous bleeding, and patients at high risk should be managed with active interventions. The current evidence suggests that an active surveillance for two years may provide the same benefits as five-year surveillance strategy, with fewer radiation hazards and less socioeconomic burden.
